# Design and Optimization of PLGA-Based Diclofenac Loaded Nanoparticles

**DOI:** 10.1371/journal.pone.0087326

**Published:** 2014-01-28

**Authors:** Dustin L. Cooper, Sam Harirforoosh

**Affiliations:** Department of Pharmaceutical Sciences, Gatton College of Pharmacy, East Tennessee State University, Johnson City, Tennessee, United States of America; Concordia University Wisconsin, United States of America

## Abstract

Drug based nanoparticle (NP) formulations have gained considerable attention over the past decade for their use in various drug formulations. NPs have been shown to increase bioavailability, decrease side effects of highly toxic drugs, and prolong drug release. Nonsteroidal anti-inflammatory drugs such as diclofenac block cyclooxygenase expression and reduce prostaglandin synthesis, which can lead to several side effects such as gastrointestinal bleeding and renal insufficiency. The aim of this study was to formulate and characterize diclofenac entrapped poly(lactide-co-glycolide) (PLGA) based nanoparticles. Nanoparticles were formulated using an emulsion-diffusion-evaporation technique with varying concentrations of poly vinyl alcohol (PVA) (0.1, 0.25, 0.5, or 1%) or didodecyldimethylammonium bromide (DMAB) (0.1, 0.25, 0.5, 0.75, or 1%) stabilizers centrifuged at 8,800 rpm or 12,000 rpm. The resultant nanoparticles were evaluated based on particle size, zeta potential, and entrapment efficacy. DMAB formulated NPs showed the lowest particle size (108±2.1 nm) and highest zeta potential (−27.71±0.6 mV) at 0.1 and 0.25% respectively, after centrifugation at 12,000 rpm. Results of the PVA based NP formulation showed the smallest particle size (92.4±7.6 nm) and highest zeta potential (−11.14±0.5 mV) at 0.25% and 1% w/v, respectively, after centrifugation at 12,000 rpm. Drug entrapment reached 77.3±3.5% and 80.2±1.2% efficiency with DMAB and PVA formulations, respectively. The results of our study indicate the use of DMAB for increased nanoparticle stability during formulation. Our study supports the effective utilization of PLGA based nanoparticle formulation for diclofenac.

## Introduction

**Figure 1:**
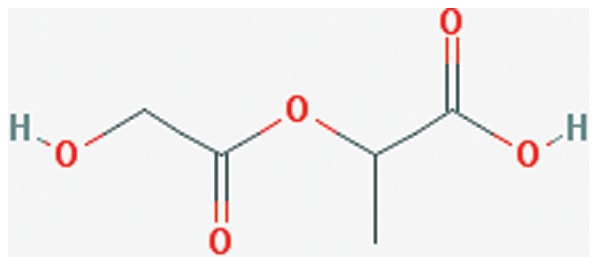
Chemical structure of poly (lactide-co-glycolide) (PLGA) [**45**].

Over the past decade, there has been an increased interest in particle manipulation and nanosizing of selected drugs. In particular, polymeric nanoparticle formulation has gained an increasing amount of public attention in the fields of drug delivery and pharmaceutics. In recent years, the application of polymer based nanoparticles in drug formulation has garnered immense attention. Industry has focused, in large part, on the utilization of biodegradable polymer based nanoparticles as effective drug delivery agents because of their ability to prolong drug release, increase drug bioavailability, decrease drug degradation and reduce drug toxicity [1]. Research in nanoparticle drug formulations has focused heavily on the use of poly(lactic acid) (PLA), poly(D,L glycolide) (PLG), and poly(lactide-co-glycolide) (PLGA) (Fig. 1) based nanoparticles because of their tissue compatibility, low toxicity, and high rate of hydrolysis [2].

**Figure 2:**
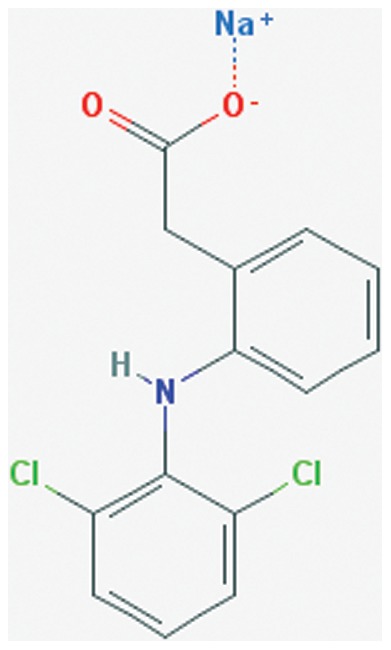
Chemical structure of diclofenac sodium [**46**].

Nonsteroidal anti-inflammatory drugs (NSAIDs) are among the most commonly prescribed drugs in the world [3]. NSAIDs are pharmaceutical agents that exert analgesic and anti-inflammatory effects through the inhibition of the cyclooxygenase family of enzymes. Diclofenac is a NSAID that is commercially available in its sodium (Fig. 2) or potassium salt form [4]. Like other NSAIDs, common side effects associated with the use of diclofenac include gastrointestinal lesion formation, and renal damage [4]. Interestingly, studies have shown a reduction in gastrointestinal and renal side effects associated with various drugs when encapsulated into polymer based nanoparticles and administered orally [5]–[7]. These results demonstrate the effectiveness of nanoparticle formulation in reducing and/or eliminating potential adverse side effects associate with orally delivered toxic drugs.

**Figure 3:**
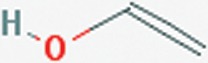
Chemical structure of poly vinyl alcohol (PVA) [**47**].

**Figure 4:**
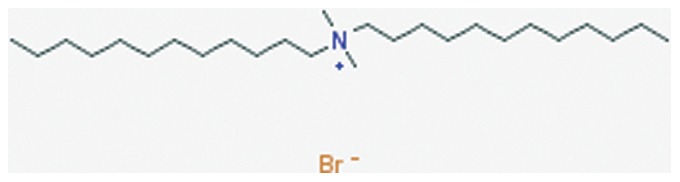
Chemical structure of didodecyldimethylammonium bromide (DMAB) [**48**].

Diclofenac nanoparticle reformulation has been used for ophthalmic and transdermal delivery with promising results [8]–[11]. The purpose of this study was to develop and characterize a new oral formulation of diclofenac using polymer based nanoparticles. Nanoparticles were synthesized using a solvent-evaporation technique and the effects of centrifugation speed and concentrations of two different stabilizers, poly (vinyl alcohol) (PVA) (Fig. 3) or didodecyldimethylammonium bromide (DMAB) (Fig. 4), was examined for effects on entrapment efficiency, particle size, and stability.

## Materials and Methods

### Materials

PLGA (50∶50 copolymer compositions; MW 30,000 Da), didodecyldimethylammonium bromide (DMAB), poly vinyl alcohol (MW 89,000 Da) and 15 mL Corning centrifuge tubes were purchased from Aldrich (St. Louis, MO, USA). Diclofenac was obtained from MP Biomedical (Solon, OH, USA). Ethyl acetate and high-performance liquidchromatography (HPLC)-grade water were purchased from Fischer Scientific Laboratory (Fair Lawn, NJ, USA). Phosphate buffer pH 7.0 was purchased from EMD Chemicals Inc. (Gibbstown, NJ, USA). 0.2 micron syringe filters were obtained from Millipore Corporation (Carrigtwohill, Ireland).

### Method of nanoparticle preparation

Nanoparticles were prepared by an emulsion – diffusion – evaporation technique [5] with slight modifications. Briefly, 45 mg of diclofenac and 50 mg of PLGA were placed in 3 mL ethyl acetate and stirred at 750 rpm for 30 minutes. Varying concentrations of PVA (0.1, 0.25, 0.5, or 1% w/v) or DMAB (0.1, 0.25, 0.5, 0.75, or 1% w/v) stabilizers were placed within 6 mL of HPLC grade water heated to 140°C and stirred at 750 rpm until fully dissolved. The organic phase was then added to aqueous phase in a drop wise manner under moderate stirring then sonicated for 5 minutes at 20 kHz using a sonic dismembrator (Fischer Scientific, Fair Lawn, NJ, USA). To facilitate diffusion, 25 mL of water was added to each emulsion under constant stirring at 750 rpm. Emulsions were stirred at 750 rpm for 4 hours to insure complete organic phase evaporation. After which, each emulsion was centrifuge (8,800 rpm or 12,000 rpm) and supernatant was collected.

### Particle size and zeta potential

Particle size was measured by dynamic light scattering using a Nicomp particle sizer (Particle Sizing Systems, Port Richy, FL, USA). Zeta potential was estimated on the basis of electrophoretic mobility under an electrical field. All measurements were performed in triplicates.

### Entrapment efficiency

To measure the amount of diclofenac nanoparticle entrapment, the amount of diclofenac present within solutions following end stage centrifugation was calculated. Diclofenac stock solution dissolved in methanol (200 mg/mL) was used to construct a standard calibration curve (10,000 – 2,000,000 ng/mL). Pure methanol was used as a blank experiment before UV measurement, after which total NP drug content was calculated using the standard curve after control for blank NPs. Quantification was performed by UV-spectrophotometry (Eppendorf Biophotometer, Hauppauge, NY, USA) with absorbance set at 280 nm. Entrapment efficiency was calculated using the following equation:

Entrapment Efficiency  =  (Amount of diclofenac entrapped within nanoparticles/Total amount of diclofenac used for synthesis) X 100

### Effects of centrifugation speed and stabilizer concentration on nanoparticle properties

NPs were formulated with five different concentrations of DMAB (0.1, 0.25, 0.5, 0.75, or 1% w/v) and four different concentrations of PVA (0.1, 0.25, 0.5, or 1% w/v). Effect of stabilizer concentrations and two centrifugation rates (8,800 or 12,000 rpm) on zeta potential, particle size, and entrapment efficiency was evaluated.

### Nanoparticle morphology characterization

Shape and surface morphology of NPs were examined with a transmission electron microscope (TEM) (Tecnai Philips Transmission Electron Microscope; FEI, Hillsboro, Oregon, USA). NP solutions were vortex mixed and 2 µL of suspension was placed on a 100 mesh copper grid covered with Formvar film (Electron Microscopy Sciences, Hatfield, Pennsylvania). Samples were kept under ventilation for 2 hours to allow for complete drying, than examined by TEM at 80 kV.

### *In vitro* drug release study

*In vitro* release of diclofenac sodium was carried out as previously described with slight modification [12], [13]. Briefly, 2 mL of solution containing diclofenac formulated nanoparticles were placed into 15 mL centrifuge tubes containing 8 mL phosphate buffer. Suspensions were then placed on an electronic shaker set at 100 rpm. At various time points, 2 mL of release medium was removed and replaced with the same volume of fresh medium. Isolated samples were centrifuged at 4,400 rpm for 5 minutes and filtered through a 0.2 micron syringe filter. Analysis was carried out using a UV spectrophotometer set at 280 nm with empty nanoparticle solutions used as control.

### Data treatment

Data is represented as mean ± standard deviation (SD). The unpaired Student's *t*-test was used to analyze cumulative release data for identical stabilizer concentrations.

## Results

### Synthesis and assembly of diclofenac loaded PLGA based nanoparticles

The synthesis of PLGA based nanoparticles was achieved through an emulsion – diffusion – evaporation technique. A solution of diclofenac and PLGA dissolved in ethyl acetate was added to an aqueous solution containing stabilizer in a drop wise manner, followed by sonication and moderate stirring for 4 hours to ensure complete organic phase evaporation. The synthesis of PLGA polymer based nanoparticles using ethyl acetate as the primary solvent has been reported before [5], [14], [15]. In the present study, PLGA NPs containing diclofenac were prepared using DMAB and PVA as stabilizers. To determine optimal nanoparticle production, varying levels of DMAB and PVA stabilizer concentration along with varying centrifugation speeds were evaluated in the determination of peak nanoparticle synthesis (Table 1). Aqueous to organic phase ratios of 1∶1 were found to elicit particle aggregation during formulation process (data not shown). As a result, a direct 1∶2 ratio of organic to aqueous phase solution was used for nanoparticle synthesis.

**Table 1:** Method of nanoparticle preparation.

	Ingredients	Amount
Organic phase	PLGA	50 mg
	Ethyl acetate	3 mL
	Diclofenac	45 mg
Aqueous phase	DMAB	Variable^1^
	PVA	Variable^2^
	HPLC-grade water	6 mL
Emulsifier	Sonic dismembrator	5 minutes (25 kHz)

1 DMAB concentrations varied 0.1, 0.25, 0.5, 0.75, and 1% w/v with respect to solvent.

2 PVA concentrations varied 0.1, 0.25, 0.5, and 1% w/v with respect to solvent.

### Influence of centrifugation and DMAB stabilizer on nanoparticle size and stability

**Figure 5:**
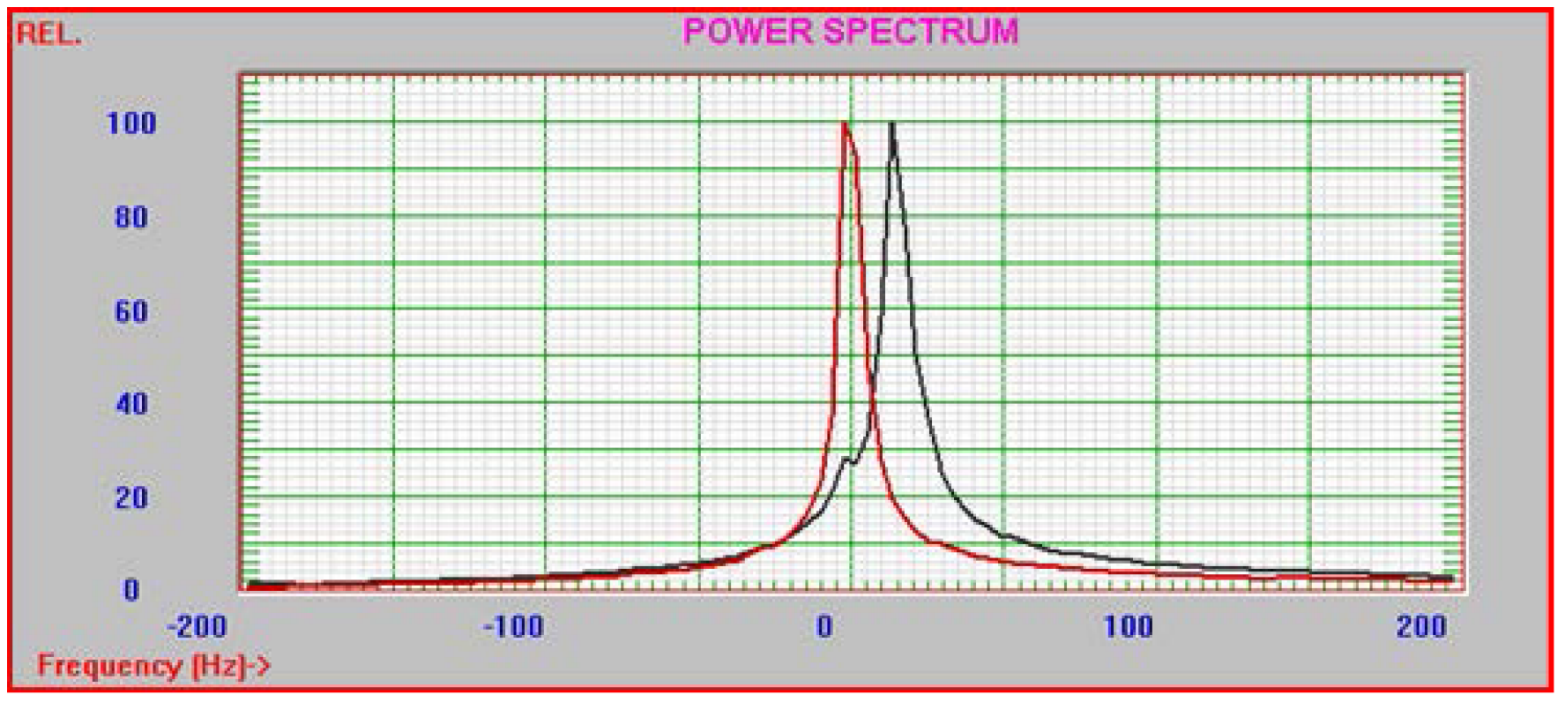
Particle Sizing Systems NICOMP analysis of diclofenac loaded 0.1% DMAB NP formulation.

Particle size and zeta potential measurements were conducted using a NICOMP Zeta Sizer System with DMAB formulated polymer NPs (Fig. 5). Measurements revealed low particle size and increased zeta potential stability with low stabilizer concentrations. Zeta potential reached peak measurements at 0.1 and 0.25% DMAB concentration. A maximum zeta potential was reached at −27.7±0.6 mV using 0.25% DMAB formulation (Table 2). Particle size was lowest using 0.1% DMAB concentrations and highest at 0.5 and 0.75% DMAB concentrations (Table 2). Interestingly, centrifugation speed was found to positively affect stability and particle size. As centrifugation speed was increased from 8,800 rpm to 12,000 rpm, there was a further increase in zeta potential and decrease in particle size when compared to lower centrifugation speed. Stability and particle size still followed the same trends as seen in lower centrifugation speeds in relation to stabilizer concentration with the exception of 0.25 and 0.5%, which showed an increase in zeta potential and reduction in particle size (Table 2).

**Table 2:** Effect of DMAB stabilizer and centrifugation speed on mean particle size and zeta potential of nanoparticles.

Centrifugation speed (rpm)	Concentration (% w/v)	Zeta potential* (mV)	Particle size* (nm)
8,800	0.1	−21.2±1.5	132.0±3.6
	0.25	−11.8±0.9	214.0±1.5
	0.5	−7.4±0.4	216.0±3.4
	0.75	−12.7±0.9	182.6±6.8
	1	Particle aggregation	Particle aggregation
12,000	0.1	−21.6±0.6	108.0±2.1
	0.25	−27.7±0.6	168.0±2.2
	0.5	−21.3±0.9	158.6±4.8
	0.75	−13.6±2.1	183.9±4.9
	1	Particle aggregation	Particle aggregation

Data are reported as mean ± SD.

* Average of triplicate measurements.

### Influence of centrifugation and PVA stabilizer on nanoparticle size and stability

Measurements of NP formulated nanoparticles using PVA stabilizer revealed lower stability and lower particle size parameters in comparison to DMAB formulations (Table 3). At 8,800 rpm centrifugation speed, particle size and zeta potential showed inverse trends in relations to stabilizer concentration. As stabilizer was increased zeta potential decreased, conversely particle size increased with increasing stabilizer concentrations. Higher centrifugation speeds maintained similar patterns with the exception of 0.25 and 1% PVA concentrations (Table 3). Formulations at 0.25% showed a slight reduction in particle size, reaching its lowest diameter at 92.4±7.6 nm. Also, 1% stabilizer formulations showed a higher degree of stability with increasing zeta potential, reaching a peak zeta potential of −11.1±0.5 mV (Table 3).

**Table 3:** Effect of PVA stabilizer and centrifugation speed on mean particle size and zeta potential of nanoparticles.

Centrifugation speed (rpm)	Concentration (% w/v)	Zeta potential* (mV)	Particle size* (nm)
8,800	0.1	−6.7±2.8	103.0±10.6
	0.25	−5.6±2.7	114.9±12.7
	0.5	−4.3±1.1	119.2±11.6
	1	−4.2±0.9	129.4±2.4
12,000	0.1	−7.4 ±0.9	94.1±12.6
	0.25	−7.0±2.1	92.4±7.6
	0.5	−4.9±2.2	113.5±22.9
	1	−11.1±0.5	120.5±6.4

Data are reported as mean ± SD.

* Average of triplicate measurements.

### Effects of stabilizer concentrations on diclofenac entrapment

**Figure 6:**
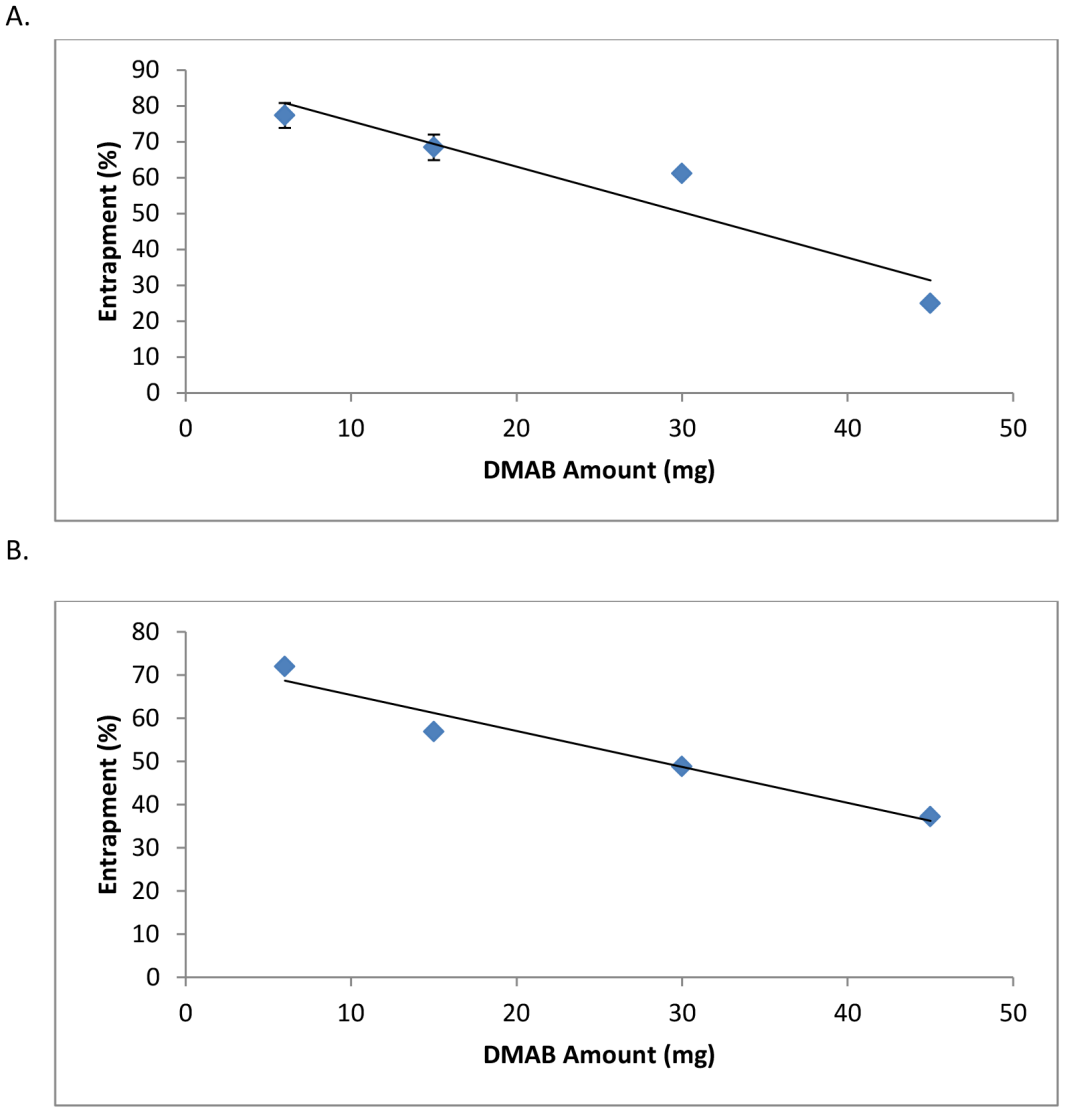
Entrapment effects of varying DMAB stabilizer concentrations. Entrapment efficiency after 8,800(A) and entrapment efficiency after 12,000 rpm centrifugation of diclofenac loaded NPs (B). Values are expressed as mean ± standard deviation.

Amount of drug entrapment was determined by UV-spectroscopy in varying stabilizer concentrations. DMAB formulated NPs reached peak entrapment at low w/v concentration. Entrapment levels with DMAB reached as high as 77.3±3.5% and were seen at 0.1% w/v DMAB concentrations. When the concentration of DMAB increased, a linear reduction in overall drug entrapment and entrapment amounts was seen (Fig. 6A) (Table 4). Conversely, as centrifugation speed was increased, slightly lower levels of drug entrapment were obtained for each formulation. Linear regression in overall drug entrapment percentages were still maintained (Fig. 6B) (Table 4).

**Table 4:** Entrapment efficiency of diclofenac loaded NPs using DMAB stabilizers at varying concentration.

Centrifugation speed (rpm)	Concentration (% w/v)	Amount entrapped* (mg)	EE* (%)
8,800	0.1	34.8±1.7	77.3±3.5
	0.25	30.8±1.6	68.4±3.6
	0.5	27.5±0.2	61.1±0.1
	1	11.2±0.1	24.9±0.1
12,000	0.1	32.4±0.2	71.9±0.4
	0.25	25.6±0.2	56.8±0.4
	0.5	21.9±0.3	48.8±0.1
	1	16.7±0.1	37.2±0.2

Amount entrapped per 45 mg diclofenac.

EE, entrapment efficiency.

Data are reported as mean ± SD.

* Average of triplicate measurements.

Measurements of drug entrapment utilizing PVA stabilizers showed similar findings to DMAB formulations. Drug entrapment levels reached 73.6±0.9% and 75.2±1.7% entrapment for PVA formulations at 0.25 and 0.5% w/v (Table 5). When centrifugation speed was increased, drug entrapment of diclofenac reached 80.2±1.2% entrapment at a lower 0.1% PVA formulation (Table 5). Increases in centrifugation speed increased drug entrapment at 0.1%, 0.25% and 1% PVA concentrations. Drug entrapment efficiency reduced from 75.2±1.7% to 28.6±1.9% in 0.5% PVA formulations when speed in centrifugation was increased (Table 5).

**Table 5:** Entrapment efficiency of diclofenac loaded NPs using PVA stabilizers at varying concentration.

Centrifugation speed (rpm)	Concentration (% w/v)	Amount entrapped* (mg)	EE* (%)
8,800	0.1	31.7±0.4	70.3±1.1
	0.25	33.1±0.4	73.6±0.9
	0.5	33.9±0.9	75.2±1.7
	1	29.9±0.6	66.4±1.2
12,000	0.1	36.1±0.5	80.2±1.2
	0.25	34.7±0.2	77.1±0.6
	0.5	14.5±0.9	28.6±1.9
	1	31.4±0.4	69.8±0.2

Amount entrapped per 45 mg diclofenac.

EE, entrapment efficiency.

Data are reported as mean ± SD.

* Average of triplicate measurements.

### Nanoparticle shape and surface morphology

**Figure 7:**
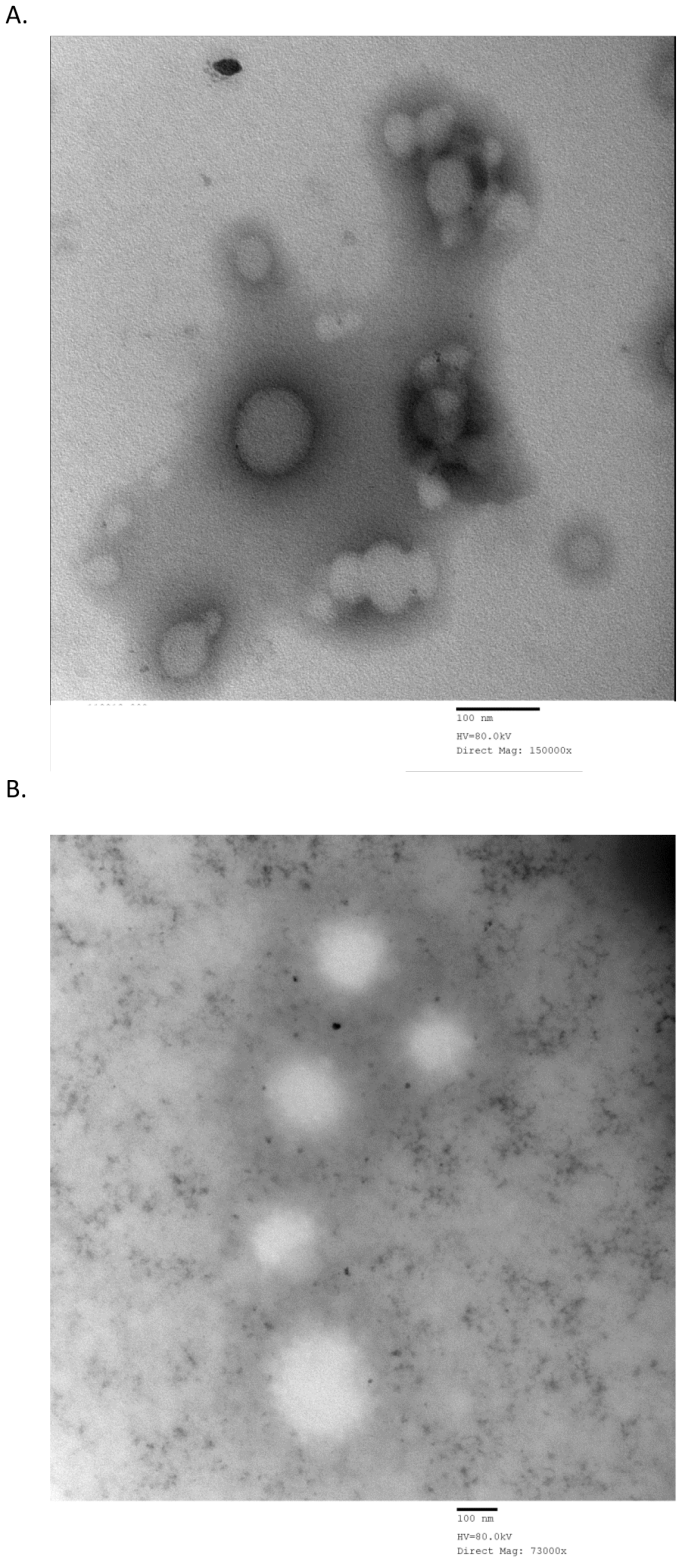
Morphological analysis of 0.25% DMAB formulated NPs. Transmission electron microscopy image of empty NPs formulated with 0.25% DMAB stabilizer (A) and transmission electron microscopy image of diclofenac loaded NPs formulated with 0.25% DMAB stabilizer (B).

**Figure 8:**
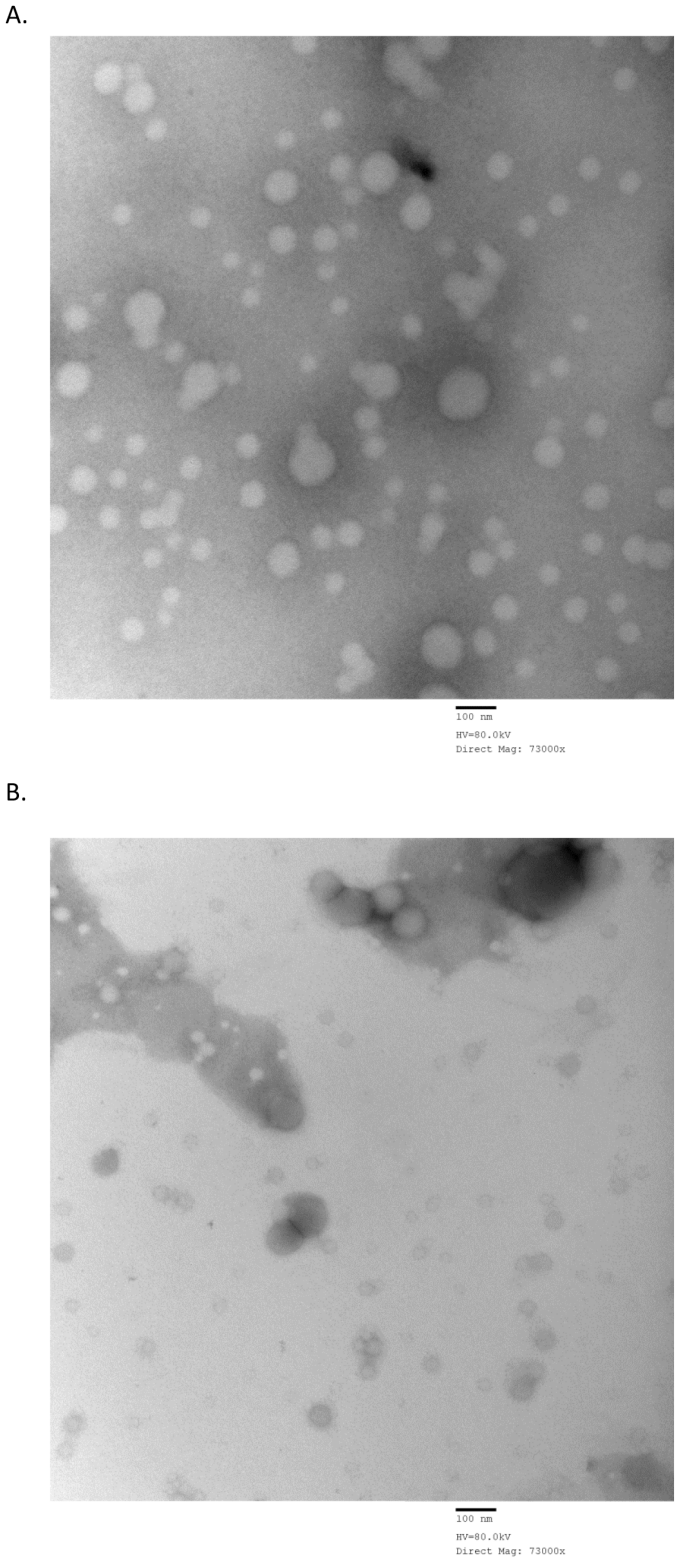
Morphological analysis of 1% PVA formulated NPs. Transmission electron microscopy image of empty NPs formulated with 1% PVA stabilizer (A) and transmission electron microscopy image of diclofenac loaded NPs formulated with 1% PVA stabilizer (B).

Morphology studies were carried out using 0.25% DMAB and 1% PVA concentrations. These stabilizer concentrations were chosen based on zeta potential and nanoparticle stability characteristics. The TEM images of blank and diclofenac loaded DMAB (Fig. 7A and 7B, respectively) and PVA (Fig. 8A and 8B, respectively) formulated NPs support the particle size data obtained by our characterization studies performed with the zetasizer. DMAB formulated NPs have a distinct, spherical shape composed of a dense core with diclofenac loaded NPs showing a slightly increased size diameter due to drug incorporation (Fig. 7B). Drug incorporation did not affect overall particle shape. Morphology of PVA formulated NPs show a high degree of shape variation and aggregation in both blank NPs (Fig. 8A) and diclofenac loaded NPs (Fig. 8B).

### Stabilizer influence on *in vitro* diclofenac release

**Figure 9:**
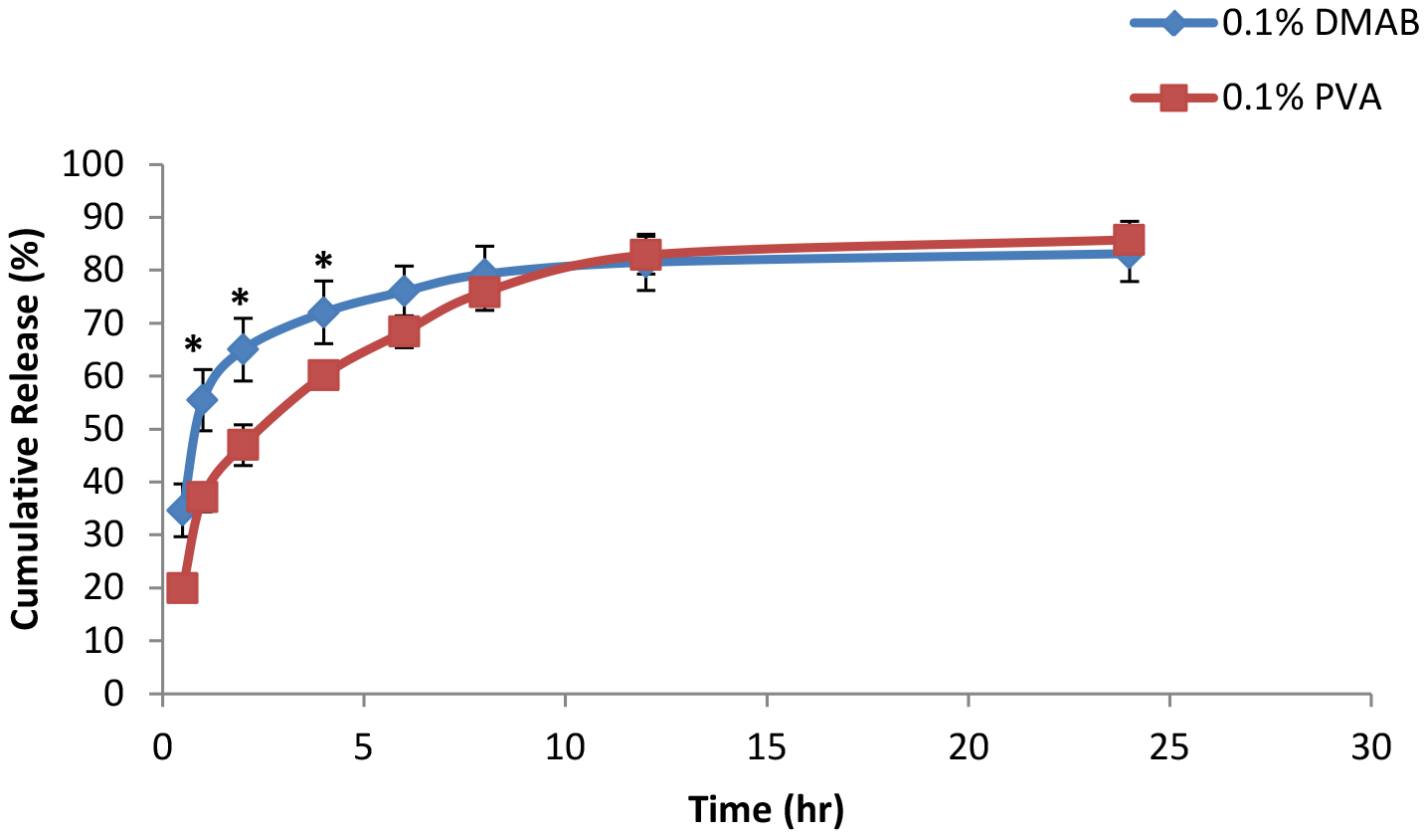
*In vitro* drug release study with 0.1% stabilizer concentrations. *In vitro* release profile of diclofenac sodium in phosphate buffer of pH 7 from 0.1% PVA formulated NPs and 0.1% DMAB formulated NPs (mean ± SD, n  =  3, p<0.05).

**Figure 10:**
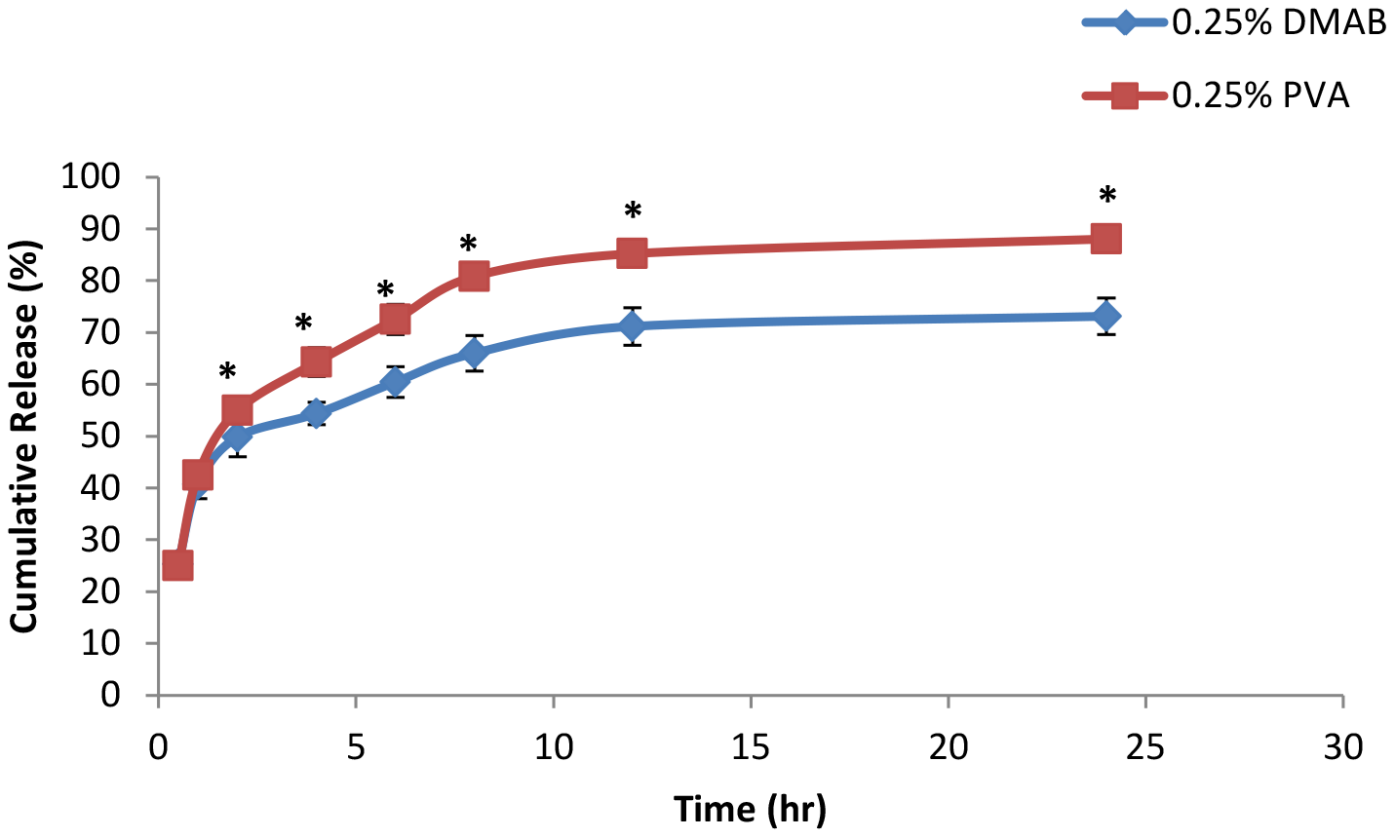
*In vitro* drug release study with 0.25% stabilizer concentrations. *In vitro* release profile of diclofenac sodium in phosphate buffer of pH 7 from 0.25% PVA formulated NPs and 0.25% DMAB formulated NPs (mean ± SD, n  =  3, p<0.05).

*In vitro* release studies were performed on two different stabilizer concentrations for both DMAB and PVA formulated NPs. Stabilizer concentrations of 0.1% and 0.25% centrifuged at 12,000 rpm were chosen based on their efficient level of drug entrapment and best fit mean representation of particle stability of each stabilizer group. The *in vitro* release of both DMAB and PVA formulated diclofenac loaded NPs are given in Figs. 9 and 10. The statistical comparison of the percentage drug release values obtained with the different nanoparticle stabilizer compositions at specific sampling times revealed significant difference (P<0.05) in both stabilizer concentration groups. DMAB formulations at 0.1% showed an initial significant increase in drug release in comparisons to 0.1% PVA formulations during the initial 4 hr time frame (Fig. 9) (P<0.05). After 24 hrs, total drug release was similar with a cumulative release of over 80% achieved for both groups (Fig. 9). The drug release of NPs formulated with 0.25% PVA showed a similar pattern of initial release of diclofenac in comparison to DMAB formulation (Fig. 10). Both formulations experienced greater than 40% release during the first hour of the study. However, after the first initial hour, cumulative release began to increase significantly in PVA formulated groups at each successive time point (P<0.05). PVA formulations reached an average cumulative release percentage of 88%, while DMAB formulation reached an average cumulative release of 73% (Fig. 10).

## Discussion

The adverse cardiovascular, gastrointestinal, and renal side effect caused by NSAID consumption has restricted the clinical use of these important drugs. The main objective of our research was to reformulate and develop a new nanoparticle formulation for diclofenac sodium that could replace traditional capsule and tablet oral administration and minimize or delay the onset of adverse side effects commonly associated with NSAIDs. Nanoparticles have been used for the development of a variety of different drug delivery systems [1], [14], [16]–[20]. Recent studies have shown the use of polymer based nanoparticles in the reformulation of diclofenac for ophthalmic and colonic us with promising results [13], [21]–[23]. Topically formulated diclofenac delivery systems have also been used with success for the treatment of a variety of inflammatory skin diseases [24]–[26]. Diclofenac delivery utilizing small lipid nanoparticles showed promising results in the realm of drug reformulation and enhanced drug delivery systems [27], [28]. Interestingly, the use of microspheres in reformulation has demonstrated enhanced drug entrapment and drug release of diclofenac [29]. One study showed that the use of Eudragit and alginate polymer systems improved drug release profiles and enhanced the physical properties of tablet compaction [30], while other studies have demonstrated a high degree of stability and morphology in microsphere development with the use of PVA [31], [32]. However, to date, reformulation characteristics of diclofenac nanoparticles for oral delivery has yet to be extensively examined.

In this study, diclofenac loaded PLGA NPs were formulated following an emulsion – diffusion – evaporation technique using DMAB or PVA as stabilizers. Stabilizers function as emulsifying agents that can offset the surface tension between organic and aqueous phases, thereby increasing drug solubility and nanoparticle encapsulation. Because of this understanding, a variation in the level of stabilizer used can equate to variations in nanoparticle characteristics during the formulation process [5], [33]–[36]. In our study, we formulated drug loaded NPs at varying levels of PVA and DMAB stabilizer concentrations to elucidate the most efficient formulation characteristics for maximum drug encapsulation, stability and size.

A study conducted by Cetin *et al.* [12] demonstrated low levels of diclofenac NP stability and entrapment efficiency when using a Eudragit®L100 and Eudragit®L100 PLGA based nanoparticle formulation with PVA as stabilizer. Consequently, they also showed that variations in polymer concentrations did not effectively alter NP characteristics to a measurable degree. Based on these findings diclofenac formulated NPs appeared to offer complications in achieving premium NP characteristics during formulation [12]. In our study, drug loaded NPs were prepared at varied DMAB (0.1, 0.25, 0.5, 0.75, or 1% w/v) and PVA (0.1, 0.25, 0.5, or 1% w/v) concentrations. Nanoparticle size was at its largest when DMAB concentrations were between 0.25, 0.5, and 0.75% w/v. Consequently, zeta potential and stability of NPs were highest when DMAB concentrations were lower (Table 2). Surprisingly, our study demonstrated diclofenac loaded NP particle sizes of 108 and 92.4 nm with DMAB and PVA, respectively. Zeta potential stability measurements reached as high as −27.7±0.6 mV in formulations using DMAB, and were substantially lower in formulation utilizing PVA stabilizers (Tables 2 and 3). These results are further supported by previously published findings in which a Eudragit®RS100 based formulation of diclofenac was used for nanoparticle characterization. It was found that variations in Eudragit concentrations effectively altered drug entrapment and particle diameter characteristics for diclofenac loaded NPs. Alterations in diclofenac to Eudragit concentrations resulted in variable measurements in particle diameter, ranging in size from 103±6 to 170±36 nm, which are consistent with the size variations of 92.4±7.6 to 216±3.4 nm found within our study [37]. Our morphological analysis showed distinct, well defined diclofenac loaded NPs when formulated with 0.25% DMAB stabilizer (Fig. 7B). The visualization of 1% PVA formulations showed distinct NP aggregation (Fig. 8A and B). These findings are consistent with particle properties of low zeta potential noticed during our characterization studies performed with the zetasizer. A more pronounced zeta potential value has a tendency to stabilize and prevent particle aggregation [38]. It is known that particles with a larger charge experience a much higher degree of repulsion from other like charged particles [38]. The high degree of particle aggregation of 1% PVA formulations are indicative of poor stability and reduced zeta potential [38], [39], which is in line with our initial findings. TEM scaling measured particle sizes within the range reported by zetasizer analysis for both formulations (Table 2 and 3). These findings suggest the use of specific DMAB concentrations in effectively formulating stable PLGA based diclofenac loaded NPs.

Entrapment efficiency is a crucial step in the characterization of an effectively formulated drug encapsulated nanoparticle. In our study, the result of drug encapsulation efficiencies with differing stabilizer concentrations and centrifugation speeds is shown in Tables 4 and 5. Our study showed high degrees of drug encapsulation for both DMAB and PVA formulations. In DMAB formulations, drug encapsulation followed a linear decline in the amount of drug entrapped in relation to the amount (Figs. 6A and 6B) or concentration (Table 4) of stabilizer used. The highest level of entrapment reached was 77.3±3.5% and was seen with DMAB concentrations of 0.1% w/v. It is important to note that stabilizing agents are important factors in determining the entrapment efficiency of lipophilic drugs. Stabilizers function by forming molecular micelles through interactions between hydrophobic portions of the stabilizers with the hydrophobic core of the NP. In other studies, it was shown that as concentrations of DMAB increases, entrapment of lipophilic drug increases in response [5]. Our findings have demonstrated the opposite in regards to entrapment, suggesting that the high polarizability of diclofenac effectively works against the micelle formation properties of DMAB resulting in a reduction in drug entrapment as DMAB concentrations increase.

Measurements of entrapment efficiency in formulations utilizing PVA as stabilizer showed similar results to those obtained with DMAB. An entrapment efficiency of 80.2±1.2% was seen at PVA concentrations of 0.1% following centrifugation at 12,000 rpm (Table 5). Interestingly, while entrapment efficiency remained high, zeta potential measurements remained close to zero, indicating low levels of stability (Table 3). Two possible explanations of our findings exist. One possibility is the presence of residual PVA. The presence of residual PVA on the nanoparticle surface has been found to mask charged groups existing on the surface of PVA formulated nanoparticle [40]. Thus, residual PVA may effectively create a shield between the nanoparticle and its surrounding medium, resulting in lower zeta potential measurements that still maintain higher levels of entrapment [40], [41]. A second possibility is the correlation between zeta potential and nanoparticle stability. Zeta potential measurements closer to zero represents a high degree of non-stability with a weak surface charge surrounding the NP. It is highly possible that NPs degrade and break during the centrifugation process, in turn causing entrapped drug to leak from the NP into the medium. The leakage of free drug into the medium could result in higher levels of spectrophotometric drug detection during entrapment studies.

Results of our *in vitro* study showed an increased initial diclofenac burst release for NPs formulated with 0.1% DMAB when compared to 0.1% PVA (Fig. 9). Inverse results were seen with stabilizer concentrations at 0.25%. Formulations with PVA at 0.25% concentration demonstrated a marked increase in drug release following one hour of agitation when compared to 0.25% DMAB formulation. Drug release from nanoparticles can occur through several means such as desorption of drug close to the surface of the nanoparticle, diffusion through the polymer matrix, or matrix erosion [2]. The fast release of diclofenac in 0.1% DMAB concentrations may be due to diclofenac polarity and increased levels of diclofenac absorbed closer to the surface of our DMAB nanoparticles [42]. The stunted release noticed in 0.25% DMAB formulation could be attributed to increased electrostatic adhesion of the drug molecules to the polymeric matrix. It has been shown that particles with larger zeta potential demonstrate higher adhesion of drug molecules to the polymeric matrix as a result of electrostatic adhesion [2]. It is possible that adhesion may be taking place within these particles that may reduce diffusion of diclofenac within the PLGA nanoparticle core after exposure to dissolution medium [42], [43].

The purpose of our study was to elucidate a novel formulation for diclofenac sodium using polymer based nanoparticles. Our results are based on NP formulations using two different stabilizers at varying concentrations at two distinct centrifugation speeds. As such, our results are limited to NP characteristics utilizing PVA and DMAB stabilizers. It is entirely possible that the use of other stabilizing agents could result in alterations of NP characteristics above what has been seen in our study.

Solvents play a critical role in the determination of NP characteristics as well. In our study, we utilized ethyl acetate as our primary organic solvent for NP preparation. Our choice of solvent was based on evidence seen in previous publications which utilized ethyl acetate in conjunction with other solvents on the determination of NP characteristics. Ethyl acetate was shown to be most effective at creating stable NPs in conjunction with the use of PLGA and DMAB as stabilizer [5], [44]. The use of differing solvents would alter pH characteristic of formulation medium. Since our focus was on the salt form of diclofenac it is possible that alteration in organic solvents could alter ionization and solubility of diclofenac sodium, leading to differences in particle size, stability and entrapment.

## Conclusions

In summary, our findings revealed the fact that diclofenac loaded PLGA NPs could be prepared utilizing low concentrations of PVA and DMAB stabilizers. Formulation was achieved through a very basic and simple evaporation - diffusion technique utilizing ethyl acetate as organic solvent. In comparisons to previous reports, the NPs of diclofenac developed in this study provided adequate diclofenac entrapment levels and showed superior levels of stability with a marked reduction in overall particle size. Diclofenac loaded PLGA NPs could be used as an alternative to existing oral delivery methods and aid in offsetting deleterious side effects common to NSAID use.
